# The Use of Xpert MTB/Rif for Active Case Finding among TB Contacts in North West Province, South Africa

**DOI:** 10.1155/2016/4282313

**Published:** 2016-07-14

**Authors:** Limakatso Lebina, Nigel Fuller, Tolu Osoba, Lesley Scott, Katlego Motlhaoleng, Modiehi Rakgokong, Pattamukkil Abraham, Ebrahim Variava, Neil Alexander Martinson

**Affiliations:** ^1^Perinatal HIV Research Unit, University of Witwatersrand, Johannesburg 2000, South Africa; ^2^Public Health, School of Medicine, University of Liverpool, Liverpool L69 7ZX, UK; ^3^Department of Molecular Medicine and Hematology, University of the Witwatersrand, Johannesburg 2000, South Africa; ^4^Department of Internal Medicine, Klerksdorp/Tshepong Hospital Complex, North West Department of Health and University of the Witwatersrand, Johannesburg 2000, South Africa; ^5^DST/NRF Centre of Excellence for Biomedical TB Research, University of the Witwatersrand, Johannesburg 2000, South Africa

## Abstract

*Introduction.* Tuberculosis is a major cause of morbidity and mortality especially in high HIV burden settings. Active case finding is one strategy to potentially reduce TB disease burden. Xpert MTB/Rif has recently been recommended for diagnosis of TB.* Methods.* Pragmatic randomized trial to compare diagnosis rate and turnaround time for laboratory testing for Xpert MTB/Rif with TB microscopy and culture in household contacts of patients recently diagnosed with TB.* Results.* 2464 household contacts enrolled into the study from 768 active TB index cases. 1068 (44%) were unable to give sputum, but 24 of these were already on TB treatment. 863 (53%) participants sputum samples were tested with smear and culture and 2.7% (23/863; CI: 1.62–3.78) were diagnosed with active TB. Xpert MTB/Rif was used in 515 (21%) participants; active TB was diagnosed in 1.6% (8/515; CI: 0.52–2.68).* Discussion and Conclusions.* Additional 31 cases were diagnosed with contact tracing of household members. When Xpert MTB/Rif is compared with culture, there is no significant difference in diagnostic yield.

## 1. Introduction

Despite recent reports of global reductions in annual TB incidence, tuberculosis (TB) remains a major public health problem with 9 million new TB cases diagnosed globally in 2013 [1]; TB is responsible for 2.4% of all deaths and is second after HIV as the leading infectious cause of mortality [2]. 78% of TB cases among HIV-infected individuals live in Africa [1]. In South Africa the TB burden is particularly severe; in 2011 annual TB incidence was 993/100,000 [3], when the estimated population HIV seroprevalence was 11% [4].

The WHO recommends active case finding for close contacts of a person with TB disease as one of the strategies for early diagnosis for TB and curbing transmission [1]. Typically, symptom screening is used to identify presumptive TB, which requires further investigation, and then using laboratory-based mycobacterial identification or chest X-rays to confirm or rule out the diagnosis is standard in many countries [5, 6]. Poor access to sensitive tests for TB such as mycobacterial culture and the prolonged duration to obtain both positive and negative culture results lead to limited use, particularly in developing countries where cost and limited laboratory infrastructure are barriers [5, 7]. Xpert MTB/Rif (Cepheid Sunnyvale, CA), a rapid point-of-care molecular test for TB that has sensitivity four times that of microscopy and can detect rifampicin resistance, was endorsed by the World Health Organization for use in endemic areas for TB diagnosis [8, 9].

Most studies of Xpert MTB/Rif have included presumptive TB as a source of both cases and noncases. Our prior experience has been that substantial proportions of contacts are found to have culture positive sputum, despite reporting no symptoms, and would therefore not be investigated further. The use of Xpert MTB/Rif in community screening or TB contact tracing for active TB cases, including those who are not presumptive TB, has not been reported on.

## 2. Methods

### 2.1. The Setting

Matlosana district is in the North West province. It is 160 km west of Johannesburg and has an estimated population of 500 000 people. It consists of Klerksdorp as the major town and three (Stilfontein, Orkney, and Hartbeesfontein) other gold mine towns. There are four townships (residential areas formerly designated for Blacks) around each of the towns, namely, Jouberton, Khuma, Kanana, and Tigane. The Matlosana Health district is serviced by one regional hospital and 16 community clinics.

### 2.2. Sampling

We conducted a pragmatic randomized trial of the use of Xpert MTB/Rif and TB microscopy and culture in diagnosing TB among household contacts of patients recently diagnosed with TB within a large implementation science program, done between 31/01/2011 and 07/06/2012. Xpert MTB/Rif was only introduced in the study in the last 7 months. The randomization into receiving smear, microscopy, and culture or GeneXpert was done by one of the administrators at the head office. The team leader would call while doing home visits to find out how the specific households were to be randomized. The Block Stratified Randomization Windows version 6.0 was used to assign each household to GeneXpert or standard smear microscopy and culture using the participant study numbers.

Both adults and children who had standard clinical diagnosis of TB in the last three months were considered eligible for enrollment in the massive active case finding study. A standard clinical diagnosis of TB included anyone with bacteriological/laboratory confirmation of TB or who had been started on TB treatment on the basis of clinical features or anyone who died in the hospital prior to getting TB treatment but had clinical features suggestive of TB. The index patient had to have been living in the Matlosana district for at least six months prior to enrollment. Index patients were approached to provide written informed consent for collection of their sociodemographic data and for the study team to make a household visit when other household contacts would be screened for TB. At households, each household member provided written consent with assent and parental/guardian coconsent for younger household members. The household members were enrolled if they slept in that house more than 2 nights a week or ate more than four meals a week or shared a living space for a cumulative 8 hours per week. Block randomization was used to assign each household to have their sputum assessed either by Xpert MTB/Rif or by the study standard of smear microscopy and liquid mycobacterial growth indicator tube (MGIT) culture (SLC).

The study team either interviewed or reviewed hospital or clinic records of the index patient to collect data on duration of symptoms; date of admission and date of discharge or death; the basis of the TB diagnosis; and their HIV status. During household visits contacts had a TB symptom screen according to WHO guidelines; spot sputum TB collected; HIV testing (finger prick-rapid test or laboratory saliva based oral test, Orasure); CD4 count test for HIV-infected individuals; and weight and height measurements. Participants with abnormal results were referred to their local clinics for further treatment.

Specimens of fresh sputum for SLC were sent to a central laboratory for testing. At the central laboratory, the specimen was decontaminated, auramine stained, and examined with fluorescence microscopy for detection of acid-fast bacilli (AFB and MGIT). MGIT-positive specimens received another Ziel Neelsen (ZN) stain to confirm the presence of mycobacterium. If the ZN stain was positive, the mycobacterium would undergo genotyping using HAIN test to confirm that it is* Mycobacterium tuberculosis* and whether it is resistant to any drugs. Trained personnel at four local clinics analyzed sputum for Xpert MTB/Rif. Specimens of fresh sputum were tested in GX IV Xpert (four cartridges) to analyze sputum samples for TB.

Data were analyzed using Statistical Analysis Software (SAS) version 9.2 to compare the two groups of the household contacts pragmatically randomized into receiving SLC and those that received Xpert testing. We report characteristics of index cases and household contacts. Categorical data frequencies and percentages were calculated with their 95% confidence interval (CI) and proportions were compared using the Chi-square test. Odds ratios were determined by univariate analysis and unadjusted odds ratio estimated after controlling for other risk factors. The active case finding study received local ethics approvals from the University of Witwatersrand and the regional hospital's and provincial research committees. Delayed procurement of Xpert in two clinics resulted in some contacts that were pragmatically randomized for Xpert MTB/Rif actually receiving SLC. Moreover, if sputum volumes were low or sputum was delivered too late in the afternoon they were also sent for SLC.

## 3. Results

In total, 768 households of 768 index TB cases were visited during the ten months (September 2011 to June 2012) when household members were pragmatically randomized to receive either SLC or Xpert MTB/Rif. Index TB cases were recruited from the local clinics (411; 53.5%) and 357 (46.5%) were in-patients from the hospital (Table 1). The vast majority of index cases were HIV-infected, 81% (623/768); 75.8% (582/768) had CD4 count results, of which 69.6% (405/582) were less than 250 cells/mm^3^. Specimens from 9 (2.5%) index patients were found to have multidrug resistant TB (MDRTB); all were from Tshepong hospital (Table 2).

The median number of household members was 2 (IQR 1–3; range 2–13) per household, and 2464 household members were enrolled; 9 were not included in analysis due to incomplete data. Among household contacts, 1086 (44%) were unable to provide a sputum specimen for TB screening tests and 863 (35%) participants' sputa were submitted to the laboratory for SLC while 515 (21%) of participants received Xpert MTB/Rif testing. Those who were not able to provide specimens included children, household members already on TB treatment, and those with an unproductive cough.

**Figure 1:**
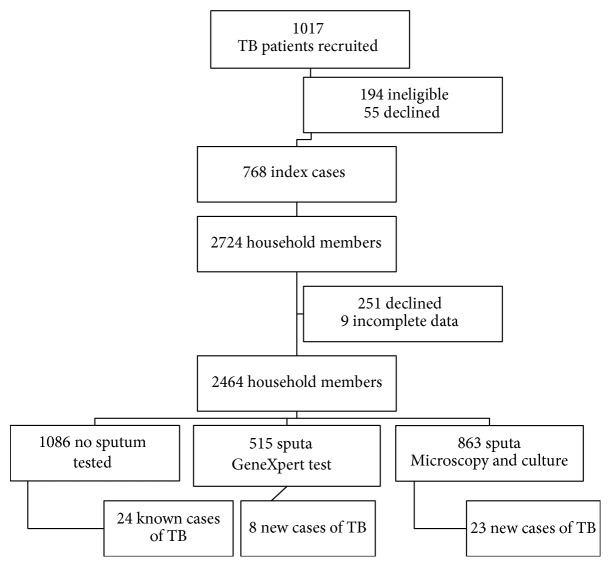
Flowchart of results of the study to determine if the use of GeneXpert is comparable to SLC in diagnosing TB among household contacts in active case finding.

Overall, based on Xpert MTB/Rif, SLC, and medical history, 55/2464 (2.2%; CI: 1.62–2.78) household members were found to have TB. A total of 24 household members were already on TB treatment based on their medical records. Therefore, 31 additional household members were diagnosed with TB by study team (1.26%; CI: 0.82–1.7). The prevalence of undiagnosed TB among the group that received SLC was 2.7% (23/863; CI: 1.62–3.78), while in Xpert MTB/Rif group it was 1.6% (8/515; CI: 0.52–2.68) (*X* ^2^ = 1.81; *P* value = 0.18) (Figure 1). All patients newly diagnosed with TB were referred to their local clinics for initiation of TB treatment.

In sputum samples submitted for SLC, 0.5% (4/863; CI: 0.01–0.91) cases of TB were diagnosed on smear alone (and confirmed on culture), and the turnaround time (laboratory testing) for smear was 2 days. Of all sputum cultures 9.4% (81/863; 7.5–11.4) of these were detected as positive by the MGIT but considered contaminated as the Ziehl Neelsen confirmation was negative in 19.8% (16/81; CI: 11.1–28.5). Further testing of the 65 sputum samples by HAIN MTBDR Plus (genotyping) confirmed* Mycobacterium tuberculosis* (MTB) in 23 cases and the other 42 cases were mycobacterium other than tuberculosis (MOTT) or contaminated. 13% (3/23; CI: −0.7–26.7) were MDRTB; 1 was resistant to only isoniazid and 1 resistant to only rifampicin.

Of the 515 samples tested by Xpert MTB/Rif, 93.2% (480/515; CI: 91–95.4) had no MTB detected, 1.6% (8/515; CI: 0.52–2.68) had MTB detected, and 5.2% (27/515; CI: 3.3–7.1) had errors or invalid results. Of the specimens in which MTB was detected, 12.5% (1/8; CI: −10.4–35.4) had rifampicin resistance detected. The error rate of results on Xpert MTB/Rif testing was 3.5% (18/515; CI: 1.9–5.1).

The majority (75.4%; 163/216; CI: 69.8–81.2) of participants that had data (*n* = 216) on the days to positivity required 15 or more days for testing to be completed on MGIT.

The TB symptoms screen was positive in 6.6% (162/2464) of the participants, with cough being the commonest symptom observed in 127 (127/162–78.4%). In the 2464 TB contacts household members that were screened, about half were symptom negative and unable to provide sputum for TB tests. There was no significant difference in positive TB screen between the Xpert MTB/Rif tested (7,7%; 40/515) and the SLC tested participants (8,6%; 74/863), *P* value 0.5985.

The overall HIV prevalence among household contacts was 16.6% (408/2464; CI: 15.1–18.1). Almost half of the participants who were unable to provide sputum had BMI below 18.5, but the same group also had about two-thirds (59.8%; 649/1086) of the participants under the age of 15 years. Only 21.6% (88/408) participants that were HIV positive had recent CD4 count results available, and 8.3% (34/408) CD4 count results were below 350 cells/mm^3^.

68.6% (1542/2247) of the participants with unknown HIV status preferred rapid HIV tests to Orasure. 77% (1730/2247) of participants who did not know their HIV status were HIV-tested; 204/1730 (11.8%) newly diagnosed HIV-infected individuals were identified and referred for further care. The risk factors for undiagnosed TB identified were HIV positive status (adjusted OR: 4.99; CI: 2.15–11.59), positive TB symptoms screen (adjusted OR: 3.13; CI: 1.2–8.17), and diabetes (adjusted OR: 3.12; CI: −0.36–26.87). However, the data on smoking did not show it to be a significant risk factor with an OR of 1.16 (CI: 0.46–2.89) in univariate analysis and an adjusted OR of 0.77 (CI: 0.22–2.76). Smokers and males also appear to have a slightly higher risk of having undiagnosed TB, but it is not significant. However when adjusting for other potentially confounding factors, male gender (OR: 2.27) and diabetes (OR: 3.12) are other additional factors with a significant risk.

## 4. Discussion

This study comparing the use of Xpert MTB/Rif and SLC in diagnosing TB among household contacts found that TB microscopy and culture diagnosed more cases of TB, but the difference in proportions was not statistically significant (*P* = 0.18). The overall undiagnosed TB prevalence in household contacts of patients recently diagnosed with TB was 1.3%.

An additional 31 cases of TB (1.3%; 31/2464; CI: 0.85–1.75) were diagnosed. This yield of new TB cases is lower than the 6% (169/2843; CI: 5–7) that was diagnosed in the same community, in another study that was comparing the prevalence of TB among household contacts with an active TB patient and random households with no known active TB case [10]. However the yield is still higher than the 0.4% (4/983; CI: 0.01–0.8) that was diagnosed in random households [10]. This also confirms that contact tracing that is targeted at community members considered to be at high risk of TB like household contacts yields more new TB cases compared to community wide approach that had a yield of 0.02% [11]. The lower diagnostic yield of Xpert MTB/Rif compared to microscopy and culture (8/515; 1.6%; CI: 0.5–2.7 versus 23/863; 2.7%; CI: 1.6–3.8) was similar to that observed in the screening of mine workers for TB in which Xpert MTB/Rif diagnosed 2.1% while culture diagnosed 2.7% [12]. The differences in diagnostic yield by Xpert MTB/Rif or culture were not statically significant in this study and in that done by Dorman et al. [13].

The major advantage of Xpert MTB/Rif is that it reduces mean time of laboratory testing for TB from 16 days of culture to two hours [14]. Although the costs of Xpert MTB/Rif are higher or comparable to culture in some settings [15] the cost benefits of the quick turnaround time for results and reduced number of visits prior to diagnosis and early initiation of treatment make it cost-effective [16, 17].

The overall (newly diagnosed and known cases on treatment) prevalence of TB among household contacts was 2.2% (55/2464; CI: 1.6–2.8). This prevalence rate is higher than the country level estimate of TB prevalence of 768/100,000 (0.77%) in 2011 [3]. There were 3 cases (3/23; 13%; CI: 0.7–26.7) of confirmed multidrug resistant (MDR) TB diagnosed on culture among the newly diagnosed TB cases, and this is higher than the national level of 1.8% MDR cases in new TB cases [1]. This could have been influenced by the 9 cases (9/768; 1.2%; CI: 0.4–2.0) of the index cases being MDR TB cases.

The error rate of results on Xpert MTB/Rif testing was 3.5% (18/515; CI: 1.9–5.1). This is similar to the error or invalid results rate of 5% observed by Van Rie et al. [7]. There were a total of 42 sputum specimens (44/863; 4.7%; CI: 3.3–6.1) that tested positive on culture, which were later confirmed as mycobacterium other than tuberculosis (MOTT), and 16 (16/863; 1.9%; CI: 1.0–2.8) contaminated. The active case finding study in a mobile HIV service had 16% (162/1011; CI: 13.7–18.3) and 4.7% (47/1011; CI: 3.3−5.9) as MOTT [18].

The HIV prevalence in TB household contacts was 16% (408/2464; CI: 15.1–18.1), which is higher than the regional (North West province) estimated HIV prevalence of 11.3% [19]. Since the 1980s, HIV has been identified as a major risk factor for developing TB, and other risk factors include malnutrition, poor socioeconomic conditions, and smoking [5]. There was no data collected on nutritional status or socioeconomic status in this study to inform if these were other risk factors. The noncommunicable diseases associated with high risk of TB are diabetes mellitus and chronic tobacco-related lung disease and regular screening is recommended to exclude subclinical TB [14]. A positive TB symptom screen being a risk factor for undiagnosed TB is in keeping with the literature. The WHO has recommended symptom screen for TB as part of routine care and active case finding [20] but symptom screen has been shown to be less sensitive in HIV positive people [21–23]. TB contacts under the age of five years have been reported to be at even a higher risk of undiagnosed TB [12]. There were few children under the age of five years, but review of contacts below 15 years did not appear to be a significant risk factor. The major risk factors for undiagnosed TB in household contacts are HIV positive status (OR: 5.1) and positive symptom screen (OR: 4.9).

## 5. Conclusion

There is no significant difference in the diagnostic yield of Xpert MTB/Rif compared to microscopy and culture. Contact tracing and active case finding for household TB contacts diagnose additional cases of TB. In communities that have a high prevalence of HIV and TB home-based screening for TB and HIV provides early diagnosis of diseases and referral for the appropriate care. A large Xpert MTB/Rif and culture comparison study among household contacts in which participants receive both tests is required to establish whether Xpert MTB/Rif sensitivity compares with culture in screening for TB.

## Figures and Tables

**Table 1:** Comparison of household contacts characteristics and results following randomization to SLC or Xpert MTB/Rif test.

Variable	Sputum SLC screened	Sputum Xpert MTB/Rif screened	*P* values (calculated from *X* ^2^)
Households	393/768 (51.2%)	198/768 (25.8%)	<0.0001^*∗*^
Index patient, *hospital*	177/357 (49.6%)	92/357 (25.8%)	<0.0001^*∗*^
Jouberton township	315/863 (36.5%)	241/515 (46.8%)	0.0002^*∗*^
Gender,* female*	524/863 (60.7%)	318/515 (61.7%)	0.7046^∧^
HIV positive	200/863 (23.2%)	90/515 (17.5%)	0.0120^*∗*^
Positive TB symptom screen	74/863 (8.6%)	40/515 (7.7%)	0.5985^∧^
Smokers	146/863 (16.9%)	76/515 (14.8%)	0.2912^∧^
Alcohol use	235/863 (27.2%)	122/515 (23.7%)	0.1466^∧^
BMI < 18.5	305/863 (35.3%)	209/515 (40.5%)	0.0516^∧^
Diabetes (>10 mmol/L)	12/863 (1.4%)	6/515 (1.2%)	0.7214^∧^
New cases of TB	23/863 (2.7%)	8/515 (1.6%)	0.1782^∧^

^*∗*^Significant; ^∧^not significant.

**Table 2:** Characteristics of household members.

Variables	Sputum SLC screened	Sputum Xpert MTB/Rif screened	No sputum provided for testing
Number (%); median (IQR)			
*Population*	863 (35%)	515 (21%)	1086 (44%)
*Median age years*	27 (16–48)	23 (13–45)	10 (4–24)
<15 years	172 (19.9%)	146 (28.3%)	649 (59.8%)
15–45 years	451 (52.2%)	234 (45.6%)	332 (30.5%)
>45 years	238 (27.6%)	117 (22.7%)	105 (9.7%)
Missing	2 (0.2%)	18 (3.5%)	
*Gender*			
Males	327 (37.9%)	184 (35.9%)	492 (45.3%)
Females	524 (60.7%)	318 (61.7%)	594 (54.7%)
Missing	12 (1.4%)	13 (2.5%)	
*Township*			
Jouberton	315 (36.5%)	241 (46.8%)	381 (35.1%)
Kanana	306 (35.5%)	92 (17.9%)	427 (39.3%)
Khuma	77 (8.9%)	63 (12.2%)	105 (9.7%)
Tigane	103 (11.9%)	98 (19.0%)	125 (11.5%)
Others	62 (7.2%)	20 (4.1%)	48 (4.4%)
*HIV status *			
HIV negative	576 (66.7%)	334 (64.8%)	696 (64.1%)
HIV positive	200 (23.2%)	90 (17.5%)	118 (10.9%)
Unknown	87 (10.1%)	91 (17.7%)	272 (25%)
*Recent CD4 count*			
Number done	66	9	13
Median	394 (276; 551)	377 (184; 503)	446 (265; 635)
Below 350	25/66 (37.9%)	4/9 (44.4%)	5/13 (38.5%)
*Symptom screen *			
Cough	59 (6.8%)	30 (5.8%)	38 (3.5%)
Productive Cough	37 (4.3%)	23 (4.5%)	19 (1.7%)
Weight loss	21 (2.4%)	9 (1.8%)	15 (1.4%)
Night sweats	12 (1.4%)	9 (1.8%)	9 (0.8%)
Unwell	13 (1.5%)	6 (1.2%)	9 (0.8%)
*Smoking history*			
None	705 (81.7%)	422 (81.9%)	948 (87.3%)
Yes	146 (16.9%)	76 (14.8%)	106 (9.8%)
Missing data	12 (1.4%)	17 (3.3%)	32 (2.9%)
*Alcohol use*			
None	616 (71.4%)	377 (73.2%)	899 (82.8%)
Yes	235 (27.2%)	122 (23.7%)	155 (14.3%)
Missing data	12 (1.4%)	16 (3.1%)	32 (2.9%)

*Body mass index*			
<18.5	305 (35.3%)	209 (40.6%)	633 (58.3%)
18.5–24.9	319 (37%)	161 (31.3%)	262 (24.1%)
25–29.9	111 (12.9%)	53 (10.3%)	60 (5.5%)
>30	107 (12.4%)	72 (14.0%)	75 (6.9%)
Missing data	21 (2.4%)	20 (3.9%)	56 (5.2%)
*Blood glucose*			
Normal	793 (91.9%)	415 (80.5%)	1021 (94.0%)
High (>10 mmol/L)	12 (1.4%)	6 (1.2%)	9 (0.8%)
Not tested	58 (6.7%)	94 (18.3%)	56 (5.2%)
